# Executive summary of the Clinical Guidelines of Pharmacotherapy for Neuropathic Pain: second edition by the Japanese Society of Pain Clinicians

**DOI:** 10.1007/s00540-018-2501-0

**Published:** 2018-05-08

**Authors:** Masahiko Sumitani, Tetsuya Sakai, Yoichi Matsuda, Hiroaki Abe, Shigeki Yamaguchi, Toyoshi Hosokawa, Sei Fukui

**Affiliations:** 1 Department of Pain and Palliative Medicine, The University of Tokyo Hospital, 7-3-1 Hongo, Bunkyo-ku, Tokyo, 113-0033 Japan; 2 Department of Pain Clinic and Anesthesia, Sasebo Kyosai Hospital, Sasebo, Japan; 3 Department of Anesthesiology and Intensive Care Medicine, Graduate School of Medicine, Osaka University, Suita, Japan; 4 Department of Anesthesiology, School of Medicine, Dokkyo Medical University, Mibu, Japan; 5 Department of Pain Management and Palliative Care Medicine, Kyoto Prefectural University of Medicine, Kyoto, Japan; 6 Pain Management Clinic, Shiga University of Medical Science Hospital, Otsu, Japan

**Keywords:** Neuropathic pain, Pharmacotherapy, Guidelines

## Abstract

Neuropathic pain has a substantial effect on quality of life (QOL). The Japanese Society of Pain Clinicians (JSPC) has developed clinical guidelines of pharmacotherapy for neuropathic pain. These guidelines offer clarity on recommendations based on both the most recent scientific evidence and expert opinions. Understanding the concept, disease entity, and burden of neuropathic pain, as well as its screening and diagnosis are important steps before starting pharmacotherapy. As well as other guidelines, the guidelines propose several lines of pharmacotherapies in a step-wise manner. To name a few different points, our guidelines propose an extract from inflamed cutaneous tissue of rabbits inoculated with vaccinia virus, which has been found to be effective for post-herpetic neuralgia in Japan, as one of the second-line drugs. When prescribing opioid analgesics, proposed as the third-line drugs, for neuropathic pain, the guidelines recommend physicians continue evaluations on either abuse or addiction. The guidelines do not recommend concomitant use of nonsteroidal anti-inflammatory drugs and acetaminophen because of lack of clinical evidence of their efficacy. If patients do not respond well to pharmacotherapy, which is prescribed in a step-wise manner, other treatment strategies should be considered to improve patients’ activities of daily living and QOL.

## Introduction

Neuropathic pain has a substantial effect on quality of life (QOL) and is associated with a high economic burden for both individuals and society. It arises from a heterogeneous group disorders that affect the peripheral and central somatosensory nervous systems. It is now regarded as a distinct clinical entity despite a large variety of causes. People with neuropathic pain may experience altered pain sensation, areas of numbness or burning, and continuous or intermittent evoked or spontaneous pain. Thus, neuropathic pain is an unpleasant sensory and emotional experience. Professional organizations, including the Japanese Society of Pain Clinicians (JSPC), and nations have developed guidelines of pharmacotherapy for neuropathic pain. Existing guidelines share some common elements, including dosing thresholds, necessity of titration, and risk mitigation strategies. However, there is considerable variability in the specific recommendations (e.g., range of dosing thresholds), audience (e.g., primary care clinicians versus specialists), use of evidence (e.g., systematic review, grading of evidence and recommendations, and role of expert opinion) and rigor of methods for addressing conflicts of interest. Most guidelines do not reflect the insurance system and approved medications in respective countries. In 2011, the JSPC published the Clinical Guidelines of Pharmacotherapy for Neuropathic Pain, first edition [1], based on evidence from scientific studies available at that time. According to the most recent scientific evidence, clinical guidelines should be updated after every 5 years. Therefore, the JSPC renewed the guidelines and published its second edition in 2016 [2]. These guidelines offered clarity on recommendations based on the most recent scientific evidence in addition to existing evidence and expert opinions. The diversity of scientific viewpoints about neuropathic pain and its pharmacological treatment strategies is still not enough. Therefore, the JSPC based the recommendations after consideration of the clinical evidence, contextual evidence (including benefits and harms, values and preferences, and resource allocation), and expert opinions. The JSPC hopes that not only Japanese, but also international intended end-users are aware of the existence of the guidelines and put them into practice. After publication, dissemination of the key messages of the guidelines is of paramount importance. Publication of executive summaries as a quick reference guide and the production in a format like as a downloadable version, which can be globally read and understood as a stand-alone document, are helpful. To accomplish this, the present article summarizes the recommendations and rationales of the Clinical Guidelines of Pharmacotherapy for Neuropathic Pain, second edition, published in 2016 (Table 1). Interested readers would be referred to the full-length version of the guidelines to completely understand the proper context of the recommendations.

**Table 1:** Clinical questions in the clinical guidelines for pharmacotherapy of neuropathic pain by the Japanese Society of Pain Clinicians (second edition)

1. Neuropathic pain
CQ1: How do we define and understand neuropathic pain in clinical medicine? *A*
2. Pathology of Neuropathic Pain
CQ2: How do we understand the pathology of neuropathic pain? *A*
3. Diseases which present with neuropathic pain
CQ3: What diseases are associated with neuropathic pain? *A*
4. Neuropathic pain classification and mixed pain condition
CQ4: What is the neuropathic and nociceptive pain classification and its clinical significance? *A*
5. Pain associated with acute peripheral nerve inflammation
CQ5: Is acute pain that is associated with peripheral nerve inflammation regarded as neuropathic pain? *2C*
6. Chronic pain syndrome and neuropathic pain
CQ6: What is the chronic pain syndrome in neuropathic pain patients? *B*
7. Epidemiology of neuropathic pain
CQ7: Are there any epidemiological surveys on the prevalence of neuropathic pain? *D*
CQ8: Are there any epidemiological surveys on the prevalence of neuropathic pain in cancer patients? *C*
8. Diagnosis of neuropathic pain
CQ9: How do we screen patients who may have neuropathic pain? *1D*
CQ10: How do we diagnose neuropathic pain? *1D*
9. Clinical characteristics of neuropathic pain
CQ11: What are the clinical characteristics of neuropathic pain? *2D*
10. Neuropathic pain and quality of life (QOL)
CQ12: What is the impact of neuropathic pain on QOL? *1B*
11. Management plan for neuropathic pain: general remarks
CQ13: What is the summary of the management plan for neuropathic pain? *B*
12. Treatment goal for neuropathic pain
CQ14: How do we establish the treatment goal for neuropathic pain? *1D*
13. What is neuropathic pain?
CQ15: What are indexes of the effects of pharmacotherapy for neuropathic pain and the levels of recommendation for the respective drugs? *1B*
13-1 First-line drugs
• Pregabalin/gabapentin
• Tricyclic antidepressants (TCAs)
• Serotonin-noradrenaline reuptake inhibitors (SNRI)
13-2 Second-line drugs
• Extract from inflamed cutaneous tissue of rabbits inoculated with vaccinia virus
• Opioid analgesic [weak]: tramadol
13-3 Third-line drugs
• Opioid analgesic
CQ16: What is the level of recommendation for NSAIDs and acetaminophen for neuropathic pain? *1B*
14. Calcium channel α-2 delta ligand
CQ17: What is the level of recommendation for pregabalin for neuropathic pain? *1A*
15. Tricyclic antidepressants
CQ18: Are tricyclic antidepressants useful for neuropathic pain? *1B*
CQ19:What are tricyclic antidepressants (TCAs)? How can we differentiate their uses? *1B*
16. Serotonin-noradrenaline reuptake inhibitor (SNRI)
CQ20: Are SNRIs effective for neuropathic pain? *1A*
17. Extracts from Inflamed Cutaneous Tissue of Rabbits Inoculated with Vaccinia Virus
CQ21: What are the features of the extracts from inflamed cutaneous tissue of rabbits inoculated with vaccinia virus? *2B*
18. Pharmacotherapy for neuropathic pain: tramadol
CQ22: What is the recommendation for tramadol for neuropathic pain? *1A*
19. Opioid analgesics [moderate]: Buprenorphine transdermal patch
CQ23: What are the features of buprenorphine? *(not applicable)*
CQ24: Is buprenorphine effective for neuropathic pain? *2C*
CQ25: What is the efficacy of the buprenorphine patch for neuropathic pain? *2C*
CQ26: What is the safety and tolerability of the buprenorphine patch? *1B*
20. Opioid analgesics [strong]:
CQ27: Are strong opioid analgesics useful for neuropathic pain? *2C*
21. Pharmacotherapy for Neuropathic pain
22. Other antidepressants
CQ28: Are anti-depressants other than TCAs and SNRIs useful for neuropathic pain? *2C*
23. Anti-epileptics
CQ29: Are anti-epileptics other than pregabalin/gabapentin more effective for neuropathic pain compared to placebo? *2C*
24. N-methyl-D-aspartate (NMDA) receptor antagonists
CQ30: Are NMDA receptor agonists useful for neuropathic pain? *2C*
25. Anti-arrhythmic drug
CQ31: Is an anti-arrhythmic drug (mexiletine hydrochloride) effective for neuropathic pain? *2B*
26. Chinese herbal medicine
CQ32: Is Chinese herbal medicine effective for neuropathic pain? *2D*
27. Post-herpetic neuralgia (chronic phase)
CQ33: What is the first drug to be considered for post-herpetic neuralgia? *1A*
CQ34: Are opioids effective for post-herpetic neuralgia? *2B*
CQ35: Is there any other drug that should be considered for post-herpetic neuralgia? *1B*
28. Post-traumatic peripheral neuropathy
CQ36: Are calcium channel alpha-2-delta ligands useful for post-traumatic peripheral neuropathy? *2B*
CQ37: Are opioids useful for post-traumatic peripheral neuropathic pain? *2C*
CQ38: Are there any other drug therapies that are effective for post-traumatic peripheral neuropathic pain? *2D*
29. Pharmacotherapy for painful diabetic neuropathy
CQ39: What is the basic management plan and the level of recommendation for drugs for painful diabetic neuropathy? *1B*
30. Trigeminal neuralgia
CQ40: Is carbamazepine more effective for trigeminal neuralgia compared to placebo? *1B*
CQ41: Are there any drugs other than carbamazepine that are effective for trigeminal neuralgia? *2C*
31. Central neuropathic pain
CQ42: What drug therapies are useful for central post-stroke pain? *2B*
CQ43: What drug therapies are useful for neuropathic pain associated with multiple sclerosis? *2C*
32. Pain after spinal cord injury
CQ44: Are TCAs and calcium channel alpha-2-delta ligands useful for pain after spinal cord injury? *1A*
CQ45:Are opioids useful for pain after spinal cord injury? *2B*
CQ46: Are there any drugs that are effective for pain after spinal cord injury other than TCAs, calcium channel alpha-2-delta ligands, and opioids? *2C*
33. Chemotherapy-induced peripheral neuropathy
CQ47: Is duloxetine useful for chemotherapy-induced peripheral neuropathy? *1C*
CQ48: Are there any drugs other than duloxetine that are useful for chemotherapy-induced peripheral neuropathy? *2D*
34. Neuropathic pain directly caused by cancer
CQ49: Are strong opioids effective for neuropathic pain directly caused by cancer? *1A*
CQ50: Are neuropathic medications effective for neuropathic pain directly caused by cancer? *2C*
35. Post-operative neuropathic pain (e.g., painful scar) and iatrogenic neuropathy (e.g., post-thoracotomy neuropathic pain, post-mastectomy neuropathic pain)
CQ51: Does perioperative drug administration reduce post-operative neuropathic pain? *1B*
CQ52: Are there any drugs that are useful for complete chronic post-thoracotomy pain? *1A*
CQ53:Are there any drugs that are useful for complete chronic post-mastectomy pain? *1B*
CQ54: What drug is useful for pain after inguinal hernia repair? *2B*
37. Cervical and lumbar radiculopathy
CQ55: Are antidepressants useful for cervical and lumbar radiculopathy? *2B*
CQ56: Are calcium channel alpha-2-delta ligands effective for lumbar radiculopathy? *1C*
CQ57: Are opioids effective for cervical and lumbar radiculopathy? *2D*
CQ58: Are there any drugs other than antidepressants, calcium channel alpha-2-delta ligands, and opioids that are effective for cervical and lumbar radiculopathy? *2D*

The guidelines are structured by three large categories (i.e., overview of neuropathic pain; diagnosis and treatment of neuropathic pain; and disease with present neuropathic pain) and 37 sub-entries. For these, 58 clinical questions (CQs) are set (details in Table 1). In this executive summary, we described headings of the sub-entries and CQs which indicate numbers in the guidelines (Table 1).

### Task force

The JSPC committee nominated the task force members from a pool of specialists with adequate clinical experience to cover multidisciplinary areas, and the JSPC Board gave the final approval. The task force comprised a total 30 physicians: 3 academic consultants, 1 external expert, 7 core working members, 8 working members, and 11 collaborators (“Appendix”).

### Drafting recommendations

A draft of clinical questions (CQs) was created by the core members. Each member in charge of the CQs drafted the recommendations and general background descriptions. Then, respective members reviewed, modified, and rewrote each statement on a reciprocal basis. In some fields, only outdated articles such as for tricyclic antidepressant were available for references. The entire articles, including the latest ones, were reviewed regardless of the published year. The reference articles included those searched under PubMed, Japan Medical Abstract Society (excluding the minutes) and Cochrane Collaboration. Finally, the external expert reviewed the statements in these guidelines, and the final version was established. The document created by each author was reviewed and revised twice in a cross-checking manner and then finally reviewed and revised again by the entire committee members.

### Evidence and recommendation levels

The task force decided to use the evidence and recommendation levels (Table 2), based on the recommendations of the Japanese Medical Information Network Distribution Service (MINDS) for developing clinical practice guidelines, which was published in 2014 by the Japan Council for Quality Health Care. Evaluations were made on all crucial outcomes, including hazard, of all important articles. The levels were first suggested by the authors and cross checked twice by the core members and then determined by the entire guidelines committee. Finally, the committee discussed the entire evidence to decide whether it can be recommended. The final levels of recommendations for each of the CQs were thus determined.

**Table 2:** Level of evidence and strength of recommendation

Level of evidence
*Level A* (*strong*) Evidence from the results of studies is established. The results will not change even if further studies are conducted
*Level B* (*moderate*) Some clinical investigations moderately support the results but evidence is not enough and confirmed. Further studies might change the results
*Level C* (*low*) Although some clinical investigations suggest the results, the results are still controversial. Further studies would be required and these might change the results
*Level D* (*very low*) There is insufficient evidence for the results. Further studies should be conducted to consider the validity of the results
Strength of recommendation
1 (strong): Recommended treatment is certainly of benefit to patients with neuropathic pain, and the benefit exceeds the harm or burden. In the statement, the term “should” is used
2 (weak): Recommended treatment might be of benefit to patients with neuropathic pain or the benefit may or may not exceed the harm or burden from the recommended treatment. In the statement, the term “might” is use

### Overview of the understanding neuropathic pain

**CQ1: How do we define and understand neuropathic pain in clinical medicine?**

**CQ2: How do we understand the pathology of neuropathic pain?**

**CQ6: What is the chronic pain syndrome in neuropathic pain patients?**

Neuropathic pain is defined as “pain caused by a lesion or disease of the somatosensory nervous system” [3]. Neuropathic pain should not indicate a single disease, but rather should be recognized as a pathological condition present in many patients complaining of pain. Neuropathic pain emerges when there is a lesion or disease in any of the nociceptive pathways from the peripheral nerves to the cerebrum. The pathological mechanisms include abnormal sensitivity of the somatosensory nervous system and functional impairment in the descending pain modulatory system. It is noteworthy that clinical criteria, which are based on overall findings of patients with neuropathic pain, are necessary in the diagnosis of neuropathic pain because it is often impossible to demonstrate consistent data from diagnostic tests for neuropathic pain. In “Guidelines for Pharmacological Treatment of Neuropathic Pain” published by the JSPC in 2011, the term “damage” had been used to describe a “lesion.” As this term “lesion” includes a condition that does not involve an irreversible anatomical change such as compression, it was changed to “lesion” according to the “Taxonomy for Pain Clinics” issued by the JSPC (2011). In addition to these biological factors, it should be mentioned that pain is usually affected by bio-psycho-social factors. Hence, we need clinical criteria that not only evaluate the pathological condition of the somatosensory nervous system, but also predict the presence or absence of psychosocial factors. In fact, neuropathic pain is accompanied by various comorbidities such as sleep disorder, lack of energy, depression, anxiety, dry mouth, and loss of appetite [4]. Although it has not been clearly understood how these comorbidities are associated with pain, the psychosocial factors for these conditions are consistent with those of a vicious cycle known as the fear-avoidance model, in addition to sleep disorder. These conditions have not yet been defined, but are referred to as the “chronic pain syndrome” [5], which is a consequence of complex interactions of bio-psycho-social factors. Therefore, we should evaluate their impact on patients’ QOL, and then determine the management plan.

### Diseases which present neuropathic pain

**CQ3: What diseases are associated with neuropathic pain?**

**CQ4: What is the neuropathic and nociceptive pain classification and its clinical significance?**

**CQ5: Is acute pain that is associated with peripheral nerve inflammation regarded as neuropathic pain?**

Nutritional, metabolic, traumatic, ischemic, toxic, genetic, infectious, compression/entrapment, immune, neoplastic, or neurodegenerative disorders can cause neuropathic pain. Table 3 lists some diseases that can be associated with neuropathic pain. These are just examples, and there are more diseases that are not listed in this table [6]. Pain is defined as “an unpleasant sensory and emotional experience associated with actual or potential tissue damage, or described in terms of such damage” [5]. The types of pain developed by bodily causes are classified into nociceptive pain and neuropathic pain. Nociceptive pain is defined as “pain that arises from actual or threatened damage to non-neural tissues and is due to the activation of nociceptors” [7]. It will be helpful to classify and evaluate nociceptive pain and neuropathic pain when we plan to treat pain due to these causes. However, pathological conditions of nociceptive pain and neuropathic pain often overlap clinically, and such a state is called the mixed pain condition. To control the mixed pain condition, pharmacotherapies for each pathologic condition would be necessary for appropriate pain control.

**Table 3:** Diseases that can cause neuropathic pain

Nutrition metabolism	Traumatic	
Alcoholic polyneuropathy Alcoholic neuropathy Neuropathy due to malnutrition (e.g., beriberi, pellagra) Hypothyroid neuropathy Painful diabetic polyneuropathy Uremic neuropathy Fabry disease Porphyric neuropathy	Iatrogenic neuropathy Post-thoracotomy pain syndrome Post-traumatic sequelae/post-operative sequelae (e.g., persistent post-operative wound pain) Post-ischemic myelopathy Phantom pain Nerve root avulsion Neuropathic myelopathy Nerve injury sequelae Tethered cord syndrome Spinal cord Hemorrhage/infarction Spinal cord injury sequelae Multiple cranial neuropathy	Stump neuralgia Post-mastectomy Stroke sequelae (e.g. thalamic pain, CNS vascular malformation) Complex Regional Pain Syndrome Post-herniorrhaphy pain Radiation-induced plexopathy Radiation-induced encephalopathy/myelopathy Peripheral neurotmesis/injury Brachial plexus avulsion
*Genetic*		
Hereditary polyneuropathy with liability to pressure palsy Hereditary sensory and autoimmune neuropathy		
*Ischemic*	*Toxic*	*Infectious*
Allergic granulomatous vasculitis Reversible ischemic neuropathy Ischemic neuropathy Connective tissue disease (vasculitis) Polyarteritis nodosa Cryoglobulinemia Mononeuritis multiplex	Chemotherapy-induced neuropathy Gold Mercurial poisoning Toxic neuromyopathy Thinner Lead Arsenic poisoning Drug-induced polyneuropathy Subacute myelo-optico neuropathy (SMON)	Diphtheritic polyneuropathy Neurosyphilis Tabes dorsalis Post-herpetic neuralgia Leprosy neuropathy Lyme disease HIV sensory neuropathy HIV myelopathy HIV neuropathy
*Compression/entrapment*		
Crural neuralgia Cervical spondylotic radiculopathy Cubital/antebrachial/wrist/foot/thigh/shoulder Entrapment neuropathy Sciatica Sciatic nerve entrapment Trigeminal neuralgia Cervical/thoracic/lumbosacral spinal cord radiculopathy Neuralgia	Carpal tunnel syndrome Cervical/lumbar spondylolisthesis Myeloradiculopathy Myelopathy Spinal canal stenosis Compressive myelopathy due to spinal canal stenosis Glossopharyngeal neuropathy Hypoglossal neuropathy Multiple sclerosis Polyneuropathy	Intervertebral disc displacement Chronic neuralgia Chronic cauda equine disorder Lumbar sciatic neuralgia Lumbar spondylosis Low back pain Intercostal neuralgia
*Immune*	*Neoplastic*	*Degenerative*
Carcinomatous neuropathy Guillain–Barre syndrome Sjogren’s syndrome Autoimmune neuropathy Plexitis Inflammatory demyelinating polyneuropathy Idiopathic neuropathy	Malignant tumor Nerve compression by tumor or neuralgia due to tumor invasion Spinal cord tumor Brain tumor Peripheral nerve tumor Neuroma Neurosarcoidosis Neurilemmoma	Amyloidotic autonomic neuropathy Charcot joint Autonomic neuropathy Syringomyelia/syringobulbia Parkinson’s disease Adrenomyeloneuropathy

There is a controversy regarding whether acute peripheral nerve inflammation should be included in the neuropathic pain category. The most representative diseases that develop acute pain in association with direct inflammation on the peripheral nerve include shingles in the acute phase and radiculopathy due to intervertebral disc displacement. Although nociceptive pain and neuropathic pain may be present at the same time during a transition phase from acute to chronic pain in association with peripheral nerve inflammation, it is currently difficult to figure out how much of the acute pain induced by shingles or intervertebral disc displacement is neuropathic pain. Therefore, in these guidelines, we would not include the acute pain associated with terminal nerve inflammation in the neuropathic pain category.

### Epidemiology of neuropathic pain

**CQ7: Are there any epidemiological surveys on the prevalence of neuropathic pain?**

**CQ8: Are there any epidemiological surveys on the prevalence of neuropathic pain in cancer patients?**

In surveys conducted in Japan that focused on chronic pain, an individual with chronic pain was defined as a person who had experienced pain with a severity of 4 or above on an 11-point numeric rating scale (NRS: 0 = no pain; 10 = worst pain imaginable) for at least twice a week for 3–6 months. As a result, the prevalence was found to be 15.2% for chronic pain in the musculoskeletal system and 26.4% for chronic pain among varied etiologies [8, 9]. Of these, the prevalence of those who were likely to have neuropathic pain according to the PainDETECT Japanese version [10] and the “Neuropathic Pain Screening Questionnaire (Japanese version)” was 6.5 and 6.4%, respectively.

Considering the relationship between neuropathic pain and the specific disease, cancer pain was systematically reviewed [11]. Among patients with cancer pain, 59.4% had nociceptive pain, 19.0% had only neuropathic pain, 20.1% had a mixture of nociceptive pain and neuropathic pain, and 1.5% had pain of unknown origin or other types of pain. In the European Association for Palliative Care, a study was conducted using the PainDETECT in 670 patients with pain out of 1051 cancer patients; according to the results, 79.7% patients had nociceptive pain, 16.9% had neuropathic pain, and 3.4% had pain of unknown origin. Compared to the patients with nociceptive pain, those with neuropathic pain required stronger opioid analgesics and adjuvant analgesics, and their performance state remained worse [12].

### Diagnosis and treatment of neuropathic pain

**CQ9: How do we screen patients who may have neuropathic pain?**

**CQ10: How do we diagnose neuropathic pain?**

**CQ11: What are the clinical characteristics of neuropathic pain?**

Neuropathic pain is distinctive pain that is different from nociceptive pain. It is characterized by spontaneous pain (continuous or intermittent) or pain induced by stimulation (allodynia, hypersensitivity) at the site supplied by the affected nerve, which is complicated by various sensory abnormalities caused by the disturbance of a nerve. The characteristic features of neuropathic pain are in the descriptions of the screening tools developed in the EU, US, and Japan (Table 4). The differences in the features of pain characteristic to each disease are presented in Table 5 [13–17]. Positive and negative findings in the somatosensory system of neuropathic pain and nociceptive pain can be useful when making a diagnosis (Table 5) [15].

**Table 4:** Comparisons among various screening tools

	ID Pain	NPQ	painDETECT	LANSS	DN4	Neuropathic pain screening tool
Stinging, prickling pain	+	+	+	+	+	+
Pain like electric shock or shooting pain	+	+	+	+	+	+
Smart or burning pain (irritation)	+	+	+	+	+	+
Tingling pain	+	+	+		+	+
Pain induced by light touch	+	+	+	+		+
Cold or freezing		+			+	
Pain induced by slight pressure			+			
Pain induced by heat or cold			+			
Pain induced by weather change		+				
Pain limited to joints	−					
Itchiness				+		
Pain pattern			+			
Pain radiating to the other areas (referred pain)			+			
Accompanied by change in the autonomic nerve	+					+
Hypo/hypersensitivity						+

**Table 5:** Differences in features between neuropathic pain and nociceptive (inflammatory) pain

		Neuropathic pain	Nociceptive (inflammatory) pain
Positive symptoms/signs	Spontaneous pain at the affected site	Present	Present
	Hypersensitive to pain against nociceptive warmth stimulation	Rare	Frequent
	Allodynia against cold stimulation	Frequent	Rare
	Increased sensory threshold against pressure stimulation and hypersensitivity to pain	Often	None
	Persistent feeling of stimulation after somatosensory stimulation	Often	Rare
	Characteristic subjective symptoms	Sudden pain, burning pain	Throbbing pain
	Pain spreading beyond the affected area	None	None
Negative symptoms/signs	Sensory disturbance in the area supplied by the affected nerve	Present	None
	Motor disturbance in the area supplied by the affected nerve	Often	None

Based on these characteristics of neuropathic pain, multiple screening tools have been developed to easily evaluate the possibility that a patient has neuropathic pain in routine medical practice. There are tools known as the Japanese neuropathic pain screening questionnaire [18], PainDETECT Japanese version [10], and Leeds assessment of neuropathic symptoms and signs (LANSS) Japanese version [19] developed in Japan. All of these are just screening tools for neuropathic pain in general clinical settings, and these demonstrate high sensitivity but moderate specificity. There is a systematic review that compared and evaluated the quality (e.g., validity, reliability) of each screening tool [20]. All tools were supported at the low evidence level; hence, “use of a screening tool should not replace a detailed clinical evaluation” as stated in the guidelines above. Therefore, it is recommended to use a screening tool available, but we should not use the result of the screening tool as a diagnosis of neuropathic pain by itself.

To diagnose neuropathic pain, the International Association for the Study of Pain (IASP) developed the flowchart-form diagnostic algorithm (grading system) [21]. The details of this algorithm are described elsewhere. Briefly, first identify the present illness and the past medical history that suggest neuropathic pain. Then, perform a sensory-disturbance evaluation in a neurological examination and a test that confirms the diagnosis of neurological lesion or a disease. It is desirable to establish a diagnosis following an algorithm by the IASP neuropathic pain specific interest group.

### Treatment strategy for neuropathic pain

**CQ12: What is the impact of neuropathic pain on QOL?**

**CQ13: What is the summary of the management plan for neuropathic pain?**

**CQ14: How do we establish the treatment goal for neuropathic pain?**

The severity of neuropathic pain is relatively higher than that of other pain conditions, and neuropathic pain affects greatly patients’ health-related QOL. Further, patients with neuropathic pain were more likely to have prolonged disease duration and more medical expenses [22]. It was found that the higher the pain severity, the lower the QOL of patients [23, 24].

The treatment goal should be planned based on both the severity of pain and their impaired activities of daily living (ADL) and QOL. In a clinical study of chronic pain, it is recommended to evaluate the following six items: intensity of pain, physical functions, mental functions, level of patients’ satisfaction, signs of adverse reactions, and adherence to the treatments [25, 26]. It is considered crucial to evaluate these factors comprehensively in clinical practice. The basic treatment strategy is a pharmacotherapy that can relieve the pain (Table 6). However, if the patients do not respond well to pharmacotherapy, which is prescribed in a stepwise manner, or when their adherence to pharmacotherapy is not adequate, neuromodulatory treatments or other interventional treatments are considered. Further, to improve the patients’ ADL and QOL, functional exercises such as rehabilitations are provided to patients so that they will be able to recover their self-efficacy. Thus, it is very important to provide inter- or multi-disciplinary treatments for neuropathic pain by combining various treatment approaches according to their bio-psycho-social factors.

**Table 6:** Clinical characteristics of drugs for neuropathic pain

Drug name	Dosage form	Type	Specific usage	Treatment period	Indications	Adverse reactions
First-line drug						
Amitriptyline	Oral drug	Tricyclic antidepressant (TCA), tertiary amine	Initial dose 10 mg/day, maximum 150 mg/day; Once daily, before bedtime; Increase by 10–25 mg every 3–7 days	6–8 weeks; the maximum tolerable dose for at least 2 weeks	Depression, peripheral neuropathy	Anti-cholinergic effect, QT prolongation, suicide risk Contraindications: glaucoma, prostate hypertrophy, cardiac diseases Less adverse events with secondary amine Attention required when used concomitantly with tramadol
Nortriptyline	Oral drug	TCA, tertiary amine			Depression	
Imipramine	Oral drug	TCA, secondary amine			Depression, enuresis	
Gabapentin	Oral drug	Calcium channel α_2_δligand	Initial dose 100–300 mg/day, maximum 3600 mg/day; 1–3 times/day; Increase by 100–300 mg every 1–7 days	In addition to 3–8 weeks of dose-escalation period, 2 more weeks at the maximum dose	Refractory epilepsy	Sleepiness, dizziness, peripheral edema, increased body weight A small dose should be used in patients with renal dysfunction
Pregabalin	Oral drug	Calcium channel α_2_δligand	Initial dose 25–150 mg/day, maximum 600 mg/day; 1–3 times/day; Increase by 25–150 mg every 3–7 days	4 weeks	Neuropathic pain, pain associated with fibromyalgia	
Duloxetine	Oral drug	Serotonin-noradrenaline reuptake inhibitor (SNRI)	Initial dose 20 mg/day maximum 60 mg/day; Once daily, after breakfast	4 weeks	Depression, diabetic neuropathy, fibromyalgia, chronic low back pain	Nausea TCA, attention required when used concomitantly with tramadol
Second-line drug						
An extract from inflamed cutaneous tissue of rabbits inoculated with vaccinia virus	Oral drug (and injection)	Non-proteinogenic physiologically active substance	4 tablets (16 unites)/day; Twice daily	4 weeks	Post-herpetic neuralgia, low back pain, cervicobrachial syndrome, scapulohumeral periarthritis, knee osteoarthritis	Nausea, sleepiness, incidence is below 0.1%, high tolerability
Tramadol/acetaminophen combination	Oral drug	Opioid + acetaminophen	Initial dose 1–4 tablets/day, maximum 8 tablets/day; 1–4 times/day	4 weeks	Chronic pain, pain after dental extraction	Nausea/vomiting, constipation, somnolence Attention required when used concomitantly with SSRI, SNRI, TCA and acetaminophen
Tramadol	Oral drug (and injection)	Opioid	Initial dose 25–100/day, maximum 400 mg/day; 1–4 times/day	4 weeks	Cancer pain, chronic pain	Nausea/vomiting, constipation, somnolence Attention required when used concomitantly with SSRI, SNRI, and TCA
Third-line drug						
Buprenorphine	Patch, suppository (and injection)	Opioid	Initial dose 5 mg/day, maximum 20 mg/day; Once in 7 days	4 weeks	Chronic pain difficult to treat with non-opioid analgesic (osteoarthritis, low back pain)	Nausea/vomiting, constipation, somnolence, respiratory control
Fentanyl	1-day patch, 3-day patch (and injection)	Opioid	Establish the initial dose by calculating from the opioid dose used before switching to the treatment. The maximum dose is 120 mg/day converted from morphine hydrochloride	4 weeks	Chronic pain and cancer pain difficult to treat with non-opioid analgesic Can be used just by switching from other opioids	Nausea/vomiting, constipation, somnolence, respiratory control
Oxycodone	Oral (and injection)	Opioid	Initial dose 10 mg/day, maximum 120 mg/day	4 weeks	Cancer pain	Nausea/vomiting, constipation, somnolence, respiratory control
Morphine	Oral, suppository (and injection)	Opioid	Initial dose 10 mg/day, maximum 120 mg/day	4 weeks	Cancer pain, chronic pain	Nausea/vomiting, constipation, somnolence, respiratory control

### Pharmacotherapy for neuropathic pain

**CQ15:What are indexes of the effects of pharmacotherapy for neuropathic pain and the levels of recommendation for the respective drugs?**

**Fig. 1:**
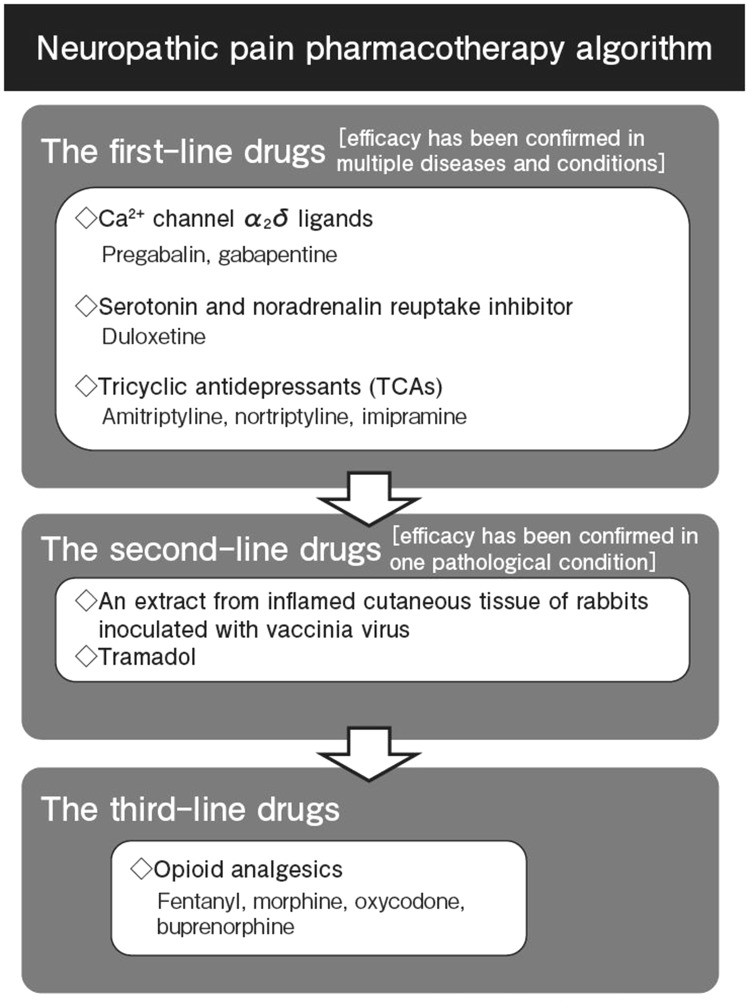
Neuropathic pain pharmacotherapy algorithm in the Japanese Society of Pain Clinicians

Pathological conditions and diseases associated with neuropathic pain vary greatly; it is extremely difficult to conduct a clinical study for each one of the conditions and diseases. Therefore, in these guidelines, we aim to present recommendations for neuropathic pain and selected drugs. Some of the drugs listed in these guidelines are not covered by the health insurance when used for neuropathic pain diseases in Japan. Such drugs should be used only after fully informing patients. Different from other published recommendations [e.g., 27], the guidelines presented an algorithm of pharmacotherapies, which is structured by the first to third-line drugs, instead of presenting an evidence list. Drugs that would have a potential to demonstrate analgesic effects on multiple diseases associated with neuropathic pain and have been approved in Japan as analgesics were selected as the first-line drugs. Out of all analgesics approved in Japan, tricyclic antidepressant (amitriptyline), pregabalin, and duloxetine are recommended as the first-line drugs. For the second-line drugs, we selected analgesic drugs that are effective for only 1 type of disease associated with neuropathic pain (Fig. 1). Opioid analgesics are shown to be effective for multiple diseases associated with neuropathic pain. However, we consider them as the third-line drugs because there have been safety concerns for their long-term use. Of all opioid analgesics, only tramadol has been classified as a second-line drug as its improvement effect on QOL is relatively high and its risk for developing addiction is low. It is desirable to receive a collaborative consultation from a pain management specialist when considering a long-term administration of opioid analgesics including tramadol.

In the original first edition of the guidelines, mexiletine hydrochloride was recommended as the second-line drug because it has been approved for painful diabetic neuropathy. However, because of low efficacy and high incidence of adverse effects, we did not recommend it in the second edition of the guidelines [27, 28]. Topical therapies with lidocaine have been reported effective [29, 30], and recommended in overseas guidelines, while this is not approved in Japan. Therefore, we did not demonstrate topical lidocaine in the algorithm.

### First-line drugs

#### Pregabalin

Pregabalin inhibits the release of excitatory neurotransmitters by combining with alpha-2-delta subunits of voltage-dependent calcium channels in the central nervous system. Similarly, gabapentin and gabapentin enacarbil work by combining with alpha-2-delta subunits of voltage-dependent calcium channels. Pregabalin is the only analgesic drug approved for both peripheral and central neuropathic pain in Japan. Neither gabapentin nor gabapentin enacarbil is approved for pain conditions. Pregabalin has greater analgesic potential compared to placebo for both Japanese and other patients with varied peripheral and central neuropathic pain diseases (e.g., post-herpetic neuralgia [31], painful diabetic polyneuropathy [32], and post-spinal cord injury pain [33]). Pregabalin also improves sleep disturbance, depression, and anxiety associated with neuropathic pain. These favorable effects can be clearly observed not only with respect to pain but also patients’ QOL.

The use of gabapentin, as well as pregabalin, for neuropathic pain is supported by evidence from studies worldwide. Gabapentin enacarbil has good evidence for neuropathic pain. However, these studies have not been confirmed for Japanese neuropathic pain patients yet.

#### Serotonin–noradrenaline reuptake inhibitors (SNRI)

The analgesic effect of serotonin-noradrenaline reuptake inhibitors (SNRIs) is considered to be mediated by activation of the descending pain inhibitory system. The analgesic potential of duloxetine, an SNRI, has been demonstrated in a clinical study on pain and numbness associated with diabetic neuropathy. Its safety has been confirmed in a 52-week study [34]. Further, it was shown that duloxetine improved central neuropathic pain compared to placebo in patients with multiple sclerosiss [35]. Duloxetine is approved in Japan for not only painful diabetic polyneuropathy and other chronic pain condition (i.e., osteoarthritis, chronic low back pain, and fibromyalgia), but also major depression. The clinical studies of duloxetine for painful diabetic polyneuropathy, osteoarthritis, and chronic low back pain did not include patients with depressive disorders in Japan. Thus, its analgesic property is independent of its anti-depressant mechanism. In addition, its analgesic effects on cancer chemotherapy-induced neuropathy [36] and low back pain associated with radiculopathy [37] have been also observed. On the basis of these evidences, duloxetine was upgraded as one of the firs-line drugs in the second edition of the guidelines from the second-line drug in the original first edition. Duloxetine improves not only pain, but also QOL in patients with peripheral neuropathy. In addition to duloxetine, two other SNRIs, venlafaxine and milnacipran, are available but both are not approved for any neuropathic pain diseases in Japan.

#### Tricyclic antidepressants (TCAs)

TCAs are significantly effective for a variety of peripheral and central neuropathic pain compared to placebo. It has been found that the analgesic mechanisms of TCAs are different from those of other anti-depressants. Among TCAs, only amitriptyline is approved for peripheral neuropathic pain in Japan. It has been reported that there is no difference in analgesic effects between the tertiary amine TCAs (amitriptyline and imipramine), which show well-balanced serotonin–noradrenaline reuptake inhibition, and the secondary amine TCA (nortriptyline), which shows relatively selective noradrenaline reuptake inhibition [38, 39]. Hence, the secondary amine TCA (nortriptyline) is considered more favorable than the tertiary amine TCAs (amitriptyline and imipramine) for being superior in tolerability, but equivalent in analgesic effects. As a majority of clinical studies using TCAs had been conducted before 2000, their effects on QOL are still unknown due to lack of appropriate evaluations.

### Second-line drugs

#### Extract from the inflamed cutaneous tissue of rabbits inoculated with vaccinia virus (ERV)

In clinical studies conducted only in Japan, the extract from inflamed cutaneous tissue of rabbits inoculated with vaccinia virus (ERV) has been found to be effective, particularly for post-herpetic neuralgia, and has been approved for it [40]. It is thought that the extract from the inflamed cutaneous tissue of rabbits inoculated with vaccinia virus activates the descending pain inhibitory system, which produces the analgesic effect. In addition to the analgesic effects, this drug does not induce serious adverse reactions and its tolerability is very high. It has been used for more than 20 years in clinical practice in Japan and has been highly safe. Although sleep disorder associated with pain improved, efficacy for other aspects of QOL has not yet been evaluated.

#### Opioid analgesic [weak]: tramadol

Tramadol acts as both a mu-opioid receptor agonist and SNRI. It is categorized as an opioid analgesic [weak], which is not designated as a restricted opioid for medical use. The analgesic effects of tramadol have been demonstrated for painful diabetic polyneuropathy [41, 42], post-herpetic neuralgia [43], and cancer-related neuropathic pain [44]. Improvement in QOL has been also confirmed. Although development of addiction is very unlikely, caution is required for long-term use. It is best to use this drug for a short-term treatment [27]. Adverse effects (e.g., constipation, sleepiness, vomiting) induced by tramadol are generally milder than those of other opioid analgesics. With both analgesic effects and QOL improvement, tramadol is given priority over other opioid analgesics. In the second edition of the guidelines, it is recommended as a second-line drug due to low safety concerns associated with long-term use [45].

### Third-line drugs

#### Opioid analgesics

Opioid analgesics are effective for a variety of diseases associated with peripheral and central neuropathic pain, including painful diabetic polyneuropathy and post-herpetic neuralgia [27]. There is abundant evidence for the analgesic efficacy of morphine and oxycodone. Transdermal fentanyl (both 1- and 3-day patches) has been approved for moderate-severe cancer pain when switching from other opioid analgesics. Buprenorphine hydrochloride is a partial agonist for mu-opioid receptors, showing equivalent efficacy. Incidence of adverse effects (e.g., nausea, constipation, sleepiness) induced by opioid analgesics is relatively high, and these could persist for a long time throughout the treatment period [46]. Moreover, there is no systematic investigation made on the long-term safety of these opioid analgesics. Opioid analgesics might not be safer than other drugs due to adverse effects, such as development of hypogonadism or addiction, though the incidence is low. We consider them as the third-line drugs because opioid analgesics have safety concerns for long-term use. Hence, it is desirable to receive a collaborative consultation from a pain management specialist when using opioid analgesics [moderate and strong] listed in this chapter. When prescribing opioids for chronic pain conditions including neuropathic pain, we always need to continue evaluations on either abuse or addiction. The recommended maintenance dose of an opioid analgesic is 15–120 mg/day when converted to oral morphine hydrochloride. The JSPC has been continuously considering the risk–benefit of pharmacotherapy for neuropathic pain from a long-term perspective. We are now debating the necessity of lowering the upper limit of maintenance doses (e.g., 60 mg/day) of opioid analgesics following a US report [47].

**CQ16: What is the level of recommendation of NSAIDs and acetaminophen for neuropathic pain?**

There is no high-quality study that has demonstrated the efficacy of both NSAIDs, including selective cyclooxygenase (COX)-2 inhibitors, and acetaminophen for neuropathic pain. Concomitant use of NSAIDs in addition to pharmacotherapy can be effective in the treatment of a mixed pain condition where neuropathic pain is complicated by nociceptive inflammatory pain [48]. It is not recommended for mixed pain condition as there is hardly any anti-inflammatory effects with acetaminophen.

## Discussion

Clinical guidelines represent one strategy for improving prescribing practices and health outcomes. Efforts are required to disseminate the guideline and achieve widespread adoption and implementation of the recommendations in clinical settings. These guidelines provide recommendations that are based on the best available evidence that was interpreted and informed by expert opinion. Some of the clinical or scientific evidence for the recommendations are still low in quality. For future guideline development worldwide, more investigations are necessary to fill in critical evidence gaps. The evidence review for these guidelines clearly illustrate that there is much to be learn about the effectiveness, safety, and economic efficiency of long-term and combined pharmacotherapies. The JSPC will revisit these guidelines as new evidence becomes available to determine when evidence gaps have been sufficiently closed to warrant an update of the clinical guidelines. These guidelines are intended to improve communication among clinicians working in varied clinical settings and patients about concept of neuropathic pain, its specific disease burden, the goals of treatment, and the risks and benefits of pharmacotherapies. These are also intended to improve the safety and effectiveness of pain treatment. The JSPC is committed to evaluating the guidelines to identify the impact of the recommendations on clinician and patient outcomes, both intended and unintended, and revising the recommendations in the future when warranted.

## Appendix

### Members of the task force for the clinical guidelines of pharmacotherapy for neuropathic pain

#### Academic consultants

Toyoshi Hosokawa, Professor, Department of Pain Management and Palliative Care Medicine, Kyoto Prefectural University of Medicine; Yasuhisa Okuda, Professor, Anesthesiology, Dokkyo Medical University Koshigaya Hospital; Kiyoshige Oseto, Professor, Department of Anesthesiology, Tokyo Medical University.

External expert.

Naoki Nogo, Director, Musashi Kokubunji Park Clinic.

#### Core working members

Sei Fukui, Clinical Professor and Chair of these guidelines, Pain Management Clinic, Shiga University of Medical Science Hospital; Hisashi Date, Director, Sendai Pain Clinic Center; Masahiko Iseki, Professor, Department of Anesthesiology and Pain Medicine, Juntendo University School of Medicine; Shigeki Yamaguchi, Professor, Department of Anesthesiology, Dokkyo Medical University School of Medicine; Masahiko Sumitani, Department Director, Department of Pain and Palliative Medicine, The University of Tokyo Hospital; Tetsuya Sakai, Associate Professor, Department of Anesthesiology, Nagasaki University Hospital; Narihito Iwashita, Lecturer, Pain Management Clinic, Shiga Medical University Hospital.

#### Working members

Jitsu Kato, Professor, Department of Anesthesiology, Division of Anesthesiology, Nihon University School of Medicine; Yoshiyuki Kimura, Associate Professor, Department of Anesthesiology, Dokkyo Medical University School of Medicine; Shizuko Kosugi, Assistant Professor, Department of Anesthesiology, Keio University School of Medicine; Munetaka Hirose, Professor, Department of Anesthesiology and Pain Medicine, Hyogo College of Medicine; Keita Fukazawa, Lecturer, Department of Pain Management and Palliative Care Medicine, Kyoto Prefectural University of Medicine; Hidekimi Fukui, Lecturer, Department of Anesthesiology, Tokyo Medical University; Yoichi Matsuda, Assistant Professor, Department of Anesthesiology and Intensive Care Medicine, Osaka University Graduate School of Medicine; Masanori Yamauchi, Professor, Department of Anesthesiology and Perioperative Medicine, Tohoku University School of Medicine.

#### Collaborators

Keisuke Yamaguchi, Associate Professor, Department of Anesthesiology and Pain Medicine, Juntendo University School of Medicine; Yoshika Takahashi, Assistant Professor, Department of Anesthesiology and Pain Medicine, Juntendo University School of Medicine; Makito Oji, Assistant Professor, Pain Clinic, NTT East Medical Center; Kumiko Hida, Assistant Professor Department of Anesthesiology, Nagasaki University School of Medicine; Koji Ishii, Assistant Professor Department of Anesthesiology, Nagasaki University School of Medicine; Keisuke Watanabe, Lecturer, Department of Anesthesiology and Pain Center, Nara Medical University; Hidekazu Watanabe, Sendai Pain Clinic Center; Noriko Taguchi, Sendai Pain Clinic Center; Tomoko Kitamura, Sendai Pain Clinic Center; Nanase Watabiki, Sendai Pain Clinic Center; Akira Yamashiro, Sendai Pain Clinic Center.
